# Interaction between cognitive styles and genders when using virtual laboratories and its influence on students of health college’s laboratory skills and cognitive load during the Corona pandemic

**DOI:** 10.1016/j.heliyon.2022.e09213

**Published:** 2022-03-30

**Authors:** Usama M. Ibrahem, Bandar S. Alsaif, Munthir Alblaihed, Sameh S.I. Ahmed, Haisam A. Alshrif, Rabab A. Abdulkader, Hanan M. Diab

**Affiliations:** a Assistant Professor, Ha’il of University, Saudi Arabia; b Educational Technology Professor, Suez Canal University, Egypt; c Educational Technology Assistant Professor, Ha’il of University, Saudi Arabia; d Educational Technology Assistant Professor, Qaboos University, Oman; e Associate Professor Curriculum and Instruction, Ha’il of University, Saudi Arabia; f Educational Technology Associate Professor, Ha’il of University, Saudi Arabia; g Educational Technology Assistant Professor, Cairo University, Egypt

**Keywords:** Virtual laboratory, Laboratory skills, Gender, General Health Colleges, Cognitive style, Cognitive load

## Abstract

This study examined the interaction between cognitive style–gender within Virtual Laboratories (VL) and its influence on students of health college’s Laboratory Skills (LS) and Cognitive Load (CL) during the Corona pandemic. This research method is a combination of quasi-experimental research and survey research; consisting of two male and two female experimental groups (contemplative and impulsive). Each group had 20 students from General Health colleges. In the third level, with the microbiological course, eight experiments were studied by a Virtual laboratory (Praxilabs) during the eLearning study in 2020’s first semester.

Results showed that VL-using Students of General Health colleges studying microbiology had better CL and LS, besides significantly distinguishing between males and females using VLs in CL and LS where males benefited more. Also, a significant difference was established between CS (contemplative/impulsive) VL-using students in CL and LS to the benefit of the contemplative cognitive style. There LS significantly differ due to gender–CS interaction; however, CL does not have any differences because of this interaction.

## 1 Introduction

An effective teaching and learning environment based on contemporary teaching tools (analogies, models, experiments, and simulations that uses ICT (such as Virtual Laboratory) improves cooperation and social interaction among students and their skills, helps them learn how to learn, and enables all students, especially those struggling with learning, while facilitating conceptual understanding (Tsihouridis et al., 2013; Ibrahem and Alamr, 2020). A Virtual Laboratory (VL) is a system that supports a conventional practicum system, providing opportunities to practice via computer and experiments are doable anywhere. A VL overcomes several laboratory-related problems and positively contributes to achieving learning purposes, especially for abstract concepts (Zaturrahmi et al., 2020; Abdjul and Nova, 2018).

VLs make students actively learn, facilitating construct and understanding difficult concepts. Furthermore, learners can overcome mistakes caused by laboratory conditions or misuse without being exposed to real laboratory conditions and dangers (Tatli and Ayas, 2012; Jolley et al., 2016). Pyatt and Sims (2012); McQueen (2017) explain that using VLs improves the lectures and laboratory in the learning process (Falode and Gambari, 2017).

Laboratory work is a form of experiential learning, where experience dominates in the process of science learning. Experience-based learning models date back to British Empiricism and John Locke, John Dewey’s philosophy of pragmatism, Jean Piaget’s theory of cognitive development, David Kolb’s experiential learning, etc, which emphasize the importance of a concrete experience, active laboratory experimentation, where learners “touch all the bases” (Bortnik et al., 2017; Ibrahem and Alamr, 2020).

An alternative learning environment, such as a VL which is becoming more popular, allows for meaningful learning. Such alternative means allow for numerous educational applications, computer-assisted physical and chemical simulations, and emulating natural phenomena and conditions of an experiment (González-Gómez et al., 2015; Seeling 2010; Tatli and Ayas 2012). After the Coronavirus, most countries proceed with their scientific career through the distance education (Saati, 2020). Distance education allowed the continuation of educational services and operations via the Internet through virtual spaces, emerging a digital generation based on modern technologies in educational processes. For example, VL allow learners to virtually access laboratories and conduct experiments, transcending teaching to visualization, perception, and simulation models (Al-Rnoussi, Al-Taie, 2016; Ibrahem and Alamro, 2021).

A CS is one of the most important personal and educational methods that individuals utilize during the educational process. It varies between those who contemplate the plausibility of the many supposed solutions in reaching an actual solution and those who respond immediately to the first opportunity or solution that comes to mind (Abu Hatab, Sadiq, 1980). Contemplation/impulsivity elucidates noncognitive aspects of personality and its behavioral effects. Al-Sharqawi (2003) defined cognitive methods as “the differences between individuals in how to practice different cognitive processes such as perception, thinking, problem-solving, and learning, as well as concerning other variables that the individual is exposed to in the behavioral situation, whether in the cognitive domain or the emotional field.” It refers to individual and distinct differences in organizing, preparing, and processing experiences.

Experimental activities enable learners to use observation to discover their concepts with thinking, problem-solving skills, and creativity. During the Corona Crisis, experimental activities were faced with many obstacles, such as lack of access to laboratory facilities and infrastructure supporting e-experimental activities; some practical activities are costly and need time to experiment with expected results. Assessing students’ performance during laboratory activities can be daunting. That is why experimental activities are not conducted. Also, from the results of observation at the Faculty of General Health in Hail of University, LS are still low, same for practicum activities besides the lack of practice measurement equipment.

In a review of empirical studies on VL, Tatli and Ayas (2012) and Bortnik et al., 2017, found a more significant improvement in VL-exposed students' performance than their conventional laboratory–exposed counterparts. Flint and Stewart (2010) stated that VLs were more cost beneficial and ten times faster than standard laboratory exercise while attaining the same learning outcomes for students who were already familiar with laboratory techniques. Tuysuz (2010) surmised that VL applications enhanced students’ achievements and attitudes unlike traditional teaching methods.

Brinson (2015) reviews recent (post 2005) empirical studies entailing sharing learning outcome achievement via traditional labs (TL, hands-on) and nontraditional labs’ (NTL, virtual, and remote) participants in the context of experimental groups. The findings illustrates that 89% of the 50 reviewed studies had students learning outcome achievement equating or exceeding NTL versus TL across all learning outcome categories (knowledge, perception, and understanding and inquiry, practical, scientific, social, and analytical skills). There was no differences between physical and virtual experiments in improved academic performance (Jolley et al., 2016; McQueen, 2017). Moreover, using e-labs in teaching and learning fails to meet the university's needs since VL are created for secondary schools. VLs appropriate for university courses are limited (Bortnik et al., 2017).

Gender issues have been linked with students' academic task performance in various studies but without any definite conclusion. Some studies showed that male students excelled over females in science courses. For instance, Kost et al. (2009) found that male students outperformed females in interactive physics, while Anagbogu and Ezeliora (2007) found that girls outperformed boys using the science process skills method of teaching. However, Adeyemi (2008) and Gambari (2010) reported that gender had no effect on academic performance. VLs help teachers keep up to date concerning technological developments, especially to streamline learning during (and after) the Covid-19 pandemic as it requires creativity to effectively deliver learning topics (Zaturrahmi et al., 2020); VL is becoming the future of experiments due to the Corona Pandemic (AL-Bedouihi, 2020; Saati, 2020) and the inevitable need for alternatives (Tuysuz, 2010; Pyatt and Sims, 2012).

Based on the previources, This article try to find answers for three Questions: the first is”What is the effect of the gender difference when using VLs on laboratory skills and cognitive load among students of public health colleges?”; the second is “What is the effect of the difference between the cognitive style (contemplative/impulsive) when using VLs on public health college students’ laboratory skills and cognitive load?”; and the third is “What is the effect of the interaction between gender and cognitive style (contemplative/impulsive) when using VLs on laboratory skills and cognitive load among public health college students?”.

## 2 Literature review

VLs allow students to interactively perform experiments in a step-by-step procedure through proper instructions and wider limitations. VLs enclose infotainment, edutainment, and enrichment with no prerequisites or fundamental knowledge of computers. VLs emulates real laboratories, displaying text, sound, graphics, videos, and animation interactively to solve real-world problems. Some are defined as computing systems enabling users to share laboratories' physical resources remotely in the absence of real laboratories or when there are no sufficient resources (Tsihouridis et al., 2013; Flowers, 2011).

### 2.1 The main tangible and intangible benefits of a VL are as follows

First, VLs are learner-centered and inquiry-based, requiring higher levels of thinking and retention, allowing students to receive immediate feedback, and rectify accordingly (Abdjul and Nova, 2018). Second, VLs are low-cost solutions for laboratory experiments. Expensive, complicated, and dangerous experiments can be simulated safely in virtual environment settings (Achuthan and Murali 2015). Third, remote labs are used as complementary tools for in-person laboratory education (Diwakar et al., 2016).

VLs enable an interactive experience where students observe and manipulate computer-generated objects, data, and phenomena to fulfill laboratory experience learning objectives. Despite being unconventional and needing minimal time for equipment setup, virtual labs are cost beneficial and can be used anytime and anywhere with swift results with no hazards. They also facilitate the repetition of the experiments with personalized learning by offering crucial feedback. While using a virtual lab, students can make mistakes without troublesome consequences. Also, VLs can graphically depict abstract ideas that may not otherwise be easily viewed, if viewed at all (Tsihouridis et al., 2013; Falode and Gambari, 2017).

VLs usage in the learning process rendered the learning process more effective and efficient and enhanced student learning achievement (Tatli and Ayas 2012). In line with that, Gunawan et al. showed that VL–assisted learning increased students' concept mastery (Gunawan et al., 2017) and improved their scientific literacy and science processing skills (Syahfitri et al., 2019) because the media provided correlated with pictures, concepts, and questions related to scientific literacy skills, even simulations of laboratory work are made as real as possible according to the concepts (Syahfitri et al., 2019; Jannati et al., 2018).

### 2.2 The cognitive style

Evidence shows that individuals maintains habitual ways of approaching tasks and situations associated with particular patterns in cognitive processes such as decision making, problem-solving, attention, and perception. Such approaches are conceptualized as a cognitive style, referring to individual differences in organizing and preparing information and experiences. It has been defined as “a trend that depends on personal preference for the steps of mental performance, as some have called it *strategies cognitive* (Bendall et al., 2016).”

### 2.3 Impulsive–contemplative style

Individuals influenced by this style tend to respond quickly in a risky manner. Often, the impulsive responses are incorrect due to the inaccurate handling of alternatives leading to the situation's solution, while individuals who tend to narrate are distinguished by examining the data in the situation while carefully approaching and verifying the alternatives before responding (Vranic et al., 2019).

### 2.4 Laboratory skills

Laboratories’ practical activities can be classified into performances requiring laboratory equipment and devices. It can be evaluated according to implementation accuracy and the speed in response to observations, as with the training or experimental situation through a method that can be improved through practice (Araby, 2004).

#### 2.4.1 Learning obstacles during Covid-19

The COVID-19 Pandemic has ceased many physical activities worldwide, including educational activities, which led to a migration to online modes of delivery to avoid hindering the learning process of students. This caused new burderns on staff and students because they had to learn to use new, compulsory software for lectures, assessments, etc. (Allen et al., 2020). For many educational institutions, the sudden shift to online learning has created an unexpected workload, especially on building e-platforms and integrating external applications into their systems promptly (Adedoyin and Soykan, 2020). Key challenges are related to technological infrastructure and digital competence, socioeconomic factors (educational inequality), heavy workload, assessment and supervision, and compatibility (Heng and Sol, 2020).

Many of them share laptops and computers with their family to stay on track, not to mention unexpected computer crashes. Also, it is hard to keep students engaged during online lectures without physical presence and face-to-face contact. Moreover, a key concern is connectivity to science labs, impossible to put into practice without in-person instructions and courses relying mostly on hands-on work (Heng and Sol, 2020). Assessment during online learning became more demanding (Adedoyin and Soykan, 2020) due to the limited control teachers have over students’ work, so it is problematic for teachers to contend with cheating.

## 3 Methods and participants

The research dealt with three variables: independent variables, VL; classification variables, gender (M–F), and CS (contemplative/impulsive); and dependent variables, C and LS. This study employed a descriptive-analytical method to analyze previous studies and a quasi-experimental transversal comparison of equivalent groups designed to identify the interaction effect of independent and classified variables on dependent variables) (The CS scale was used to classify the experimental groups as four equal experimental groups), as in Table 1:

**Table 1:** Experimental design for research.

Experimental	Treatment	Gender ∗ CS	Post Test
Group 1	VL	20 Male/contemplative	LS list + CL Scale
Group 2		20 Female/contemplative	
Group 3		20 Male/impulsive	
Group 4		20 Female/impulsive	

## 4 Procedures

### 4.1 Tools

The study have three tools, two for dada collect, and the third is exprmental tool. First, the LS list, A measuring tool including items of five-point Likert type response format was used to assess students’ LS. It comprised 20 items. Researchers initially developed the scale. The items were rated over five points, therefore providing a minimum score of 20 and a maximum of 100 points. The Likert scale has 5 categories of answer choices, namely poor level, acceptable level, good level, excellent level, and mastery level.

The reliability studies of the original form of the LS list were conducted with 40 students, and the Cronbach Alpha reliability coefficient of the test, which was found to be 0.081. Using factor analysis as evidence for content validity, the list was deemed as one-dimensional. In this research’s pilot study, the LS list was applied to 40 students, and the Cronbach alpha reliability coefficient was 0.74, whereas in the main study reliability coefficients were 0.78 for the post test.

**Second, CL scale,** Research and CL scales were examined (Al-Sharqawi, 2003, Cheon and Grant, 2012, Ibrahem et al., 2020, Kalyuga and Sweller, 2005). Then, the CL scale was prepared, initially including 20 positive and 10 negative items, and four levels of assessment were determined from the student's point of view.

To verify the scale's authenticity, arbitrators weighed it. Agreed-upon phrases were within 80–100%. The stability coefficient was calculated by the Alpha Cronbach Coefficient (α). The items’ correlation was as in Table 2:

**Table 2:** Results of the α stability coefficient for the CL Scale.

Stability Coefficient	No. of Items	No. of a Pilot Samples	Value
Cronbach Coefficient (α)	25	42	0.84

Table 2 shows that the CL instrument has high reliability (i.e., 0.84) with a total of 25 items.

**Third, Praxilabs as a VL,** Praxilabs is an application that provides virtual practicum. Based on the results of previous research, Praxilabs enables students to comprehend concepts, receive feedback, provide an interactive, constructive approach, and train students to think creatively and critically. It is available at https://praxilabs.com/ar.

### 4.2 Implementation

The program components were defined according to the scientific foundations. Then, they were presented to a group of experts in curricula and teaching methods, educational technology, and microbiology curriculum; to assess VL’s suitability for students and distance education, the goals for suitability of its content and components—besides their organization, were as follows:

1 The general objective of the program was to teach LS, identified in 20 skills.
2 The foundations of the program were grading from easy to difficult, considering individual differences in learning speed and appropriate implementation capabilities, stimulating motivations and availing interesting, enjoyable, and exciting VL content for learners.
3 Program content included the following: an introduction to VLs and how to use them, target LS and how to perform skills, information, and knowledge about laboratory experiments, and, finally, exercising laboratory experiments inside a complete simulation environment remotely.
4 The subject of learning has been identified; these Biochemistry–Pharmacology/Toxicology experiments were selected:
  ⁃ XTT ViabVility Assay
  ⁃ In Vitro Cell Viability by the Lactate Dehydrogenase Assay (LDH)
  ⁃ In Vitro Cell Viability by the Alamar Blue Assay
  ⁃ In Vitro Mammalian Cells COMET Assay-Single Cell Gel Electrophoresis (SCGE) Assay
  ⁃ In Vitro Histone H2AX Phosphorylation Assay
  ⁃ In Vitro 80HdG DNA Adduct Assay
  ⁃ In Vitro Bromodeoxyuridin (BrdU) Assay
  ⁃ In Vitro Annexin V Binding/Propidium Iodine Uptake Assay
5 Program implementation capabilities were identified by knowing the possibility of entry for students and suitable computers with them.
6 The educational method was defined as the approved VL-based distance learning method.
7 The general framework was as follows: The practical program was implemented through microbiology lessons according to the study plan within eight weeks.
8 To evaluate the program content, LS list and CL scale were used.
9 An exploratory experiment was conducted with the help of researchers in the faculty on a sample of ten students from outside the research sample and four doctors to train the assistants on conducting tests and experiment with the VL and determine its suitability to achieve learning objectives. The researchers deemed VL easy to use by learners and faculty members. The tasks are explained as follows:
  • Tasks to be performed before the learning process through the use of the VL: they included clarifying operation and interaction methods, observing learner performance, following up their integration in the VL interaction, rectifying, guiding them to the correct performance, and answering questions.
  • Tasks that have been identified for learners were a set time to enter the VL for the session, allowing training at any other suitable times.

#### 4.2.1 Implementation procedures

**Figure 1:**
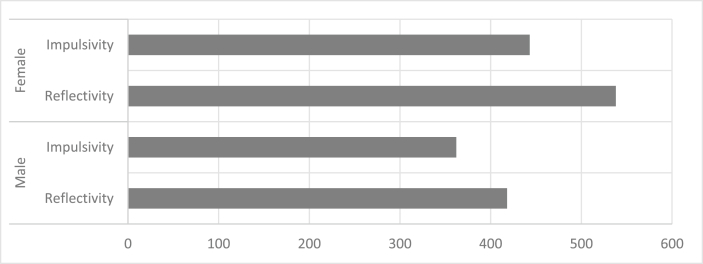
Comparison of the number of hours spent in VL by group.

- Training students on how to deal with VLs and their most important advantages in the learning process through training for 2 h, ensuring their understanding.
- There were four experimental groups according to the experimental design. Every student was given a username and password.
- When starting the application, each student enters a PDF file explaining the experience he will perform, the most important knowledge and skills to be acquired and verified, and the goal of the laboratory experiment.
- The teacher's roles were following up the application process and assisting students when needed.
- When students finished their experiment, they went to the blackboard room to answer any questions they had.
- The experimental groups were taught at the rate of one session per week for each group. The number of classes reached was 9 classes for each group within 9 weeks.
- Post measurement was as follows: after the experiment, the CL scale and LS list were applied, the total of 1761 h spent by each group in the virtual laboratory were reviewed, with each’s group hours illustrated separately in Figure 1.

The female–contemplative experimental group spent more time in the virtual laboratory by 30.6%, followed by the female–impulsive group by 25.1%, the male–contemplative group by 23.7%, and finally male–impulsive group by 20.6%. It is indicated that girls were more interested in the VL more than males, regardless of the CS.

## 5 Results and discussion

### 5.1 Limitations

Despite VLs’ positive results improving LS, there are limitations: Not all the experiment class activities were recorded. And there are patterns of interaction, discussion, and other interesting events that cannot be fully analyzed. Several difficulties appear when using VLs: (a) recommended computer standards, (b) required staff, and (c) instructors who are experts in the learning model.

### 5.2 Results

- Descriptive results

(A) Cognitive Load Levels After Using VL

The results are shown in Table 3:

**Table 3:** Metadata of CL levels after using VLs.

CL	Very High	High	Medium	Low	Very Low
Degree	25–50	51–70	71–80	81–90	91–100
No. Of Students	0	0	6	50	24
Percentage	0%	0%	7.5%	62.5%	30%

Table 4 shows that the levels of CL after using LV ranged from medium to very low. The low level came with the highest value, reaching 62.5%, while the very low level came second, reaching 30%. The average level came in the last place with a rate of 7.5%, which indicates the great effect of VLs on reducing students' CL.

(B) Laboratory Skill Levels After Using VL

**Table 4:** Metadata of laboratory skill levels after using VLs.

LS	Poor Level	Acceptable Level	Good Level	Excellent Level	Mastery Level
Degree	20–60	65–79	80–89	90–95	96–100
No. Of Students	0	2	35	38	5
Percentage	0%	2.5%	43.75%	47.5%	6.25%

Table 4 shows the metadata of LS levels after using VL:

Table 5 shows that LS levels after using VL ranged between acceptable–mastery levels. The excellent level came in the first place with a rate of 47.5%, the good level second with a rate of 43.75%, and the mastery level 6.25%, where the percentage of acceptable was 2.5%. This indicates the great effect of VL in increasing the skills levels of students with this version: This indicates the great effect of VL in improving students' skills levels.

**Table 5:** The Significance of the differences between the mean scores in LS observation list and the CL scale based on sex.

Variable	Group	No.	Average	SD.	df.	T-Value	Sig.
LS scale	M	40	89.33	7.043	78	5.967	0.000
	F	40	81.55	4.278			
CL List	M	40	90.33	2.576	78	5.217	0.000
	F	40	86.20	4.286			

The descriptive results are consistent with the studies of Syahfitri et al. (2019); Abdjul, and Nova (2018); McQueen (2017); Falode and Gambari (2017) who emphasized the role of VLs in developing science processes and skills and increasing motivation and desire for the lectures and laboratory in the learning process.

- *First hypotheses Test*

To test the first hypothesis, “There are no statistically significant differences at the level of 0.05 between the mean scores of students of Colleges of Public Health in the post-application observation card of teacher skills and the CL scale due to the main effect of the difference of sex”. A T-test enabled the determination of the significance of the differences between the mean scores of students of the College of Public Health in the post-application of the LS observation list and the CL scale based on sex. The outcomes are illustrated in Table 5:

From Table 5, the level of significance was equal to 0.000, a statistically significant value at the level of significance 0.05 for the LS observation list. The calculated value of t was 5.967. Also, the average scores for males in the post-application were 89.33, and the average scores for females in the post-application were 81.55. It also appears that the level of significance was equal to 0.000, a statistically significant value at the level of significance 0.05 for the CL scale. The value of t calculated was 5.217. The average score for males was 90.33, and the average score for females was 86.20, which indicates the superiority of males over females in the post-application of the LS observation list and the CL scale.

The first statistical hypothesis was rejected, and the alternative hypothesis was accepted, which states that “There are statistically significant differences at the level of 0.05 between the mean scores of students of the College of Public Health in the post-application of LS observation list and CL scale based on sex”. This hypothesis worked for the benefit of males when using VLs.

The results are consistent with the studies of Kost et al. (2009); Jolley et al. (2016) who emphasized that male students performed better than females when using VLs. However, it differs from the results of the studies of Anagbogu and Ezeliora (2007); Adeyemi (2008); Gambari (2010) who emphasized the performance of female students bested males when developing skills using VLs.

- *Second Hypotheses Test*

To test the second hypothesis, a T-test was used to determine the significance of the differences between the mean scores of students of the College of Public Health for the LS observation list and the CL scale based on CS differences (contemplative/impulsive). The results were reached in Table 6:

**Table 6:** The significance of the differences between the mean scores of the LS observation list and the CL scale according to the difference in the CS (Contemplative/Impulsive).

Variable	Group	No.	Average	SD.	Df.	T-Value	Sig.
LS scale	contemplative	40	89.20	6.884	78	5.693	0.000
	Impulsive	40	81.68	4.741			
CL List	contemplative	40	89.43	4.101	78	2.643	0.010
	Impulsive	40	87.10	3.761			

From Table 7, the significance level was 0.000, a statistically significant value at the level of significance 0.05 for the LS observation list, where the calculated value of t was 5.693. Also, the average grades of students with the ​contemplative ​cognitive style in the post-application reached 89.20. The average scores of students with the impulsive CS were 81.68. The level of significance was equal to 0.010, a statistically significant value at the level of significance 0.05 for the CL scale. The value of t was 2.643, and the average grades of students with the contemplative CS in the post-application were 89.43. The average grades of students with the impulsive cognitive method were metered in the post-application were 87.10.

**Table 7:** A Two-Way ANOVA analysis for the students' scores for LS observation list and the CL scale according to gender–CS interaction.

Variable	Source of Variance	Sum of Squares	Degrees of Freedom	Average Squares	F-Value	Level of Significance
LS observation List	Gender	296.450	1	296.450	66.559	0.000
	CS	273.800	1	273.800	61.474	0.000
	Gender∗ CS	68.450	1	68.450	15.368	0.000
	Error	338.500	76	4.454		
	Total	1444798	80			
CL Scale	Gender	340.313	1	340.313	29.881	0.000
	CS	108.113	1	108.113	9.493	0.003
	Gender∗ CS	1.513	1	1.513	0.133	0.717
	Error	865.550	76	11.389		
	Total	624537	80			

The results indicated that students with a contemplative CS had the highest scores in the post-application of the LS observation list and CL scale. So, the second statistical hypothesis was rejected, and the alternative hypothesis was accepted, which states “There are statistically significant differences at the level of 0.05 between the mean scores of students of General Health colleges in the post-application of the LS observation list and the CL scale based on CS differences (contemplative/impulsive) in favor of the contemplative cognitive method".

The previous results are in line with the results of Gunawan et al. (2019); Alneyadi (2019); Abou Faour and Ayoubi (2017); Pyatt and Sims (2012) who confirmed that VLs increased students’ scientific knowledge, process, and skills and intellectual abilities and attitudes, innovation.

- *To test Third Hypotheses*

We used a two-way ANOVA Analysis to test the significance of the differences between the mean differences of the LS observation list and the CL scale as in Table 7:

Table 7 shows that for the CL scale, F was 0.133 and statistical significance 0.717, which was not statistically significant at the level of significance less than 0.05.

As for the LS observation list, F was 15.368 and statistical significance 0.000, which was a statistically significant function at the level of significance less than 0.05. To determine the direction of these differences, Table 8 averages, and standard deviations were extrapolated for the post-application of the LS Observation list in light of the research variables (gender/CS).

**Table 8:** Means and standard deviations for the post-application of the LS observation list in light of the research variables (Gender/CS).

Variables			CS		Means
			Contemplative	Impulse	
Gender	Male	Mean	95.00	83.65	89.33
		Stan. dev.	2.920	5.050	7.043
	Female	Mean	83.40	79.70	81.55
		Stan. dev.	4.235	3.526	4.278

The higher average favored contemplative-cognitive males, reaching 95.00. The average for males with an impulsive-cognitive style came in second place with an average of 83.65, while the average scores of female students with a contemplative cognitive style came in third place with an average of 83.40. Finally, the female students with an impulsive-cognitive style came in an average of 79.70.

Thus, the third hypothesis was partially rejected, and the alternative hypothesis was accepted, which states that “There are statistically significant differences at levels less than 0.05 between the mean scores of health colleges’ students in the post-application of the LS observation list based on gender–CS interactions. And there are no statistically significant differences at levels less than 0.05 between the mean scores of students of General Health colleges in the post-application of the CL scale based on gender–CS interactions.

These results are unique because previous research did not address variables related to interactions in general (especially gender–CS interactions) and the effect of VL on the CS (contemplative/impulsive) on LS and CL.

### 5.3 Discussion of findings

The results show that students taught practical microbiology via VLs performed well, agreeing with the previous findings of Tatli and Ayas (2012) who established that VLs enhanced the performance of students compared with those using conventional laboratories. The finding is also supported by Tuysuz (2010); Alneyadi (2019) who found that VL applications improved achievements and attitudes.

General Health college students who studied their microbiology course through VLs had better CL and LS. Results established a significant difference between male and female students in CL and LS using VL, where males outperformed. Also, a significant difference was established between CS (contemplative/impulsive) students in CL and LS using VLs, where contemplative CS outperformed. There are significant differences in the LS based on gender–CS interaction. There are no significant differences in the CL based on gender–CS interactions.

Overall, the results of the research can be interpreted according to the Connectivism Theory. VLs’ integration was used to improve the student-centered learning process and the learner's positive role in acquiring, analyzing, practicing and exercising, and testing knowledge and skills (Alzain, 2019). The results also go along with the assumptions of Constructivist Theory, claiming that the learner constructs knowledge, learning is a personal interpretation of experience and an active process presented through real-world contexts (Hung and Yuen, 2010).

VL activity-based strategy piques students’ interest. It inflicts no stress because virtual materials, facilities, and equipment are readily available. These factors could improve students’ performance. VLs facilitated a reinforcement that avoids unpleasant experiences and reaches the highest degree of symmetry with the model without fear of errors or financial or technical costs. Also, VLs increased response time and awareness and reduced errors, where students worked without fear of failure with the chances of re-experimenting, thus reducing their CL.

Although the deliberate individual is analytical, they divide the stimuli into their component. They contemplate the group of alternative solutions before choosing a solution. So, their mistakes are reduced. In contrast, the impulsive, non-analytical individual responds quickly and behaves in a trial-and-error behavior without contemplating the alternative solutions. Hence, the impulsive individual commits many mistakes. Nevertheless, the virtual laboratory allowed impulsive students to practice and improve their mistakes to reach a good level of skills.

## 6 Conclusion and recommendations

From the above findings, it can be surmised that VLs yielded a more positive effect on students learning outcomes. It is gender-friendly and improves students’ CL and LS. Therefore, this strategy is a better approach for teaching practical skills at General Health colleges. Through VL strategy, practical content can be delivered in the simplest and most motivating and interactive manner.

These results also agree with previous research on the importance of VL's role to improve achievement, LS, and creative abilities. Universities must use many lessons learned during the period of forced adaptation to distance education to enhance and expand online learning provisions. This shift will be driven by the universities' investment in distance education and the accumulating familiarity of the students, staff, and institutions with e-learning and its visual tools.

The findings explain the novelty of the research results and the advantages of this study because it explained the interaction between cognitive style–gender within (VL) and its influence on students of health college’s (LS) and (CL) during the Corona pandemic. These results can be used in learning management in any future societal disasters, and educational designers can also use them to produce courses that provide better learning opportunities and reduce the cognitive load of learning in health colleges (which many previous studies have indicated), and give time for learners to manage their learning in the performance of advanced tasks.

Additional studies may provide light on other skills that can be improved for VL learning. It would be fascinating to look into student response patterns, various problem-solving abilities, and gender-evaluated outcomes. Problems with learning implementation time must be strictly regulated so that learning occurs according to the plans and objectives established. COVID-19 has, in some ways, prompted a rethinking of education. In that situation, there is an opportunity to conduct numerous searches for long-term improvements in the proper direction and to develop new instruments for online student learning assessment.

Based on the major findings of this study, the following recommendations are proffered:

- The CS must consider learners during the instructional design process for the VL so that there are appropriate paths for each style.
- VLs cannot replace traditional laboratories but can respond to the existing challenges and optimize the learning process.
- VL Implementation in medical education and science literacy is vital.
- Teachers should expose to VL strategy to promote active and discovery learning, motivation, and learning by doing.
- Students can benefit from VLs when learning about the real world, as they acquire conceptual knowledge and develop science process skills.

## Compliance with ethical standards

• We have no conflicts of interest to disclose.
• The research was applied to students (during their learning), and did not include Animal experimentation (so we did nor need to approval from committee).

## Declarations

### Author contribution statement

Bander S. Alsaif: Analyzed and interpreted the data; Wrote the paper.

Usama M. Ibrahem: Conceived and designed the experiments; Performed the experiments; Wrote the paper.

Munthir Alblaihed: Analyzed and interpreted the data.

Sameh S. I. Ahmed: Conceived and designed the experiments.

Haisam A. Alshrif: Contributed reagents, materials, analysis tools or data.

Rabab A. Abdulkader: Performed the experiments.

Hanan M. Diab: Contributed reagents, materials, analysis tools or data.

### Funding statement

This work was supported by The Scientific Research Deanship at the UOH, Hail, KSA Through project. (No. RG-20205).

### Data availability statement

No data was used for the research described in the article.

### Declaration of interests statement

The authors declare no conflict of interest.

### Additional information

No additional information is available for this paper.
